# Design and Evaluation of a Cochlear Implant Strategy Based on a “Phantom” Channel

**DOI:** 10.1371/journal.pone.0120148

**Published:** 2015-03-25

**Authors:** Waldo Nogueira, Leonid M. Litvak, Aniket A. Saoji, Andreas Büchner

**Affiliations:** 1 Department of Otolaryngology, Medical University Hannover, Cluster of Excellence “Hearing4all”, Hannover, Germany; 2 Research and Technology Group, Advanced Bionics LLC, Valencia CA, USA; University of South Florida, UNITED STATES

## Abstract

Unbalanced bipolar stimulation, delivered using charge balanced pulses, was used to produce “Phantom stimulation”, stimulation beyond the most apical contact of a cochlear implant’s electrode array. The Phantom channel was allocated audio frequencies below 300Hz in a speech coding strategy, conveying energy some two octaves lower than the clinical strategy and hence delivering the fundamental frequency of speech and of many musical tones. A group of 12 Advanced Bionics cochlear implant recipients took part in a chronic study investigating the fitting of the Phantom strategy and speech and music perception when using Phantom. The evaluation of speech in noise was performed immediately after fitting Phantom for the first time (Session 1) and after one month of take-home experience (Session 2). A repeated measures of analysis of variance (ANOVA) within factors strategy (Clinical, Phantom) and interaction time (Session 1, Session 2) revealed a significant effect for the interaction time and strategy. Phantom obtained a significant improvement in speech intelligibility after one month of use. Furthermore, a trend towards a better performance with Phantom (48%) with respect to F120 (37%) after 1 month of use failed to reach significance after type 1 error correction. Questionnaire results show a preference for Phantom when listening to music, likely driven by an improved balance between high and low frequencies.

## Introduction

The minimum acceptable telephone bandwidth specified in the Comité Consultatif International Télégraphique et Teléphonique (CCITT) requires lower and upper cutoff frequencies of 300 Hz and 3,400 Hz respectively [1]. This bandwidth was determined using subjective listening tests. However, listening experiments have shown that an increase of the acoustic bandwidth significantly improved not only the perceived speech quality but also speech intelligibility [2, 3].

Cochlear implant (CI) processors from Advanced Bionics have been designed to only encode spectral information above 250 Hz, mimicking the bandwidth used in telephony. Sound processors manufactured by Cochlear only transmit signals above 188 Hz in their default configuration. MED-EL processors encode down to 100 Hz by default, extendable to 70 Hz. Recent evidence in the CI field suggests also that speech cues provided by frequencies below 300 Hz can improve implant outcomes [4, 5]. In combined electrical and acoustic hearing (EAS), a CI and a hearing instrument (HI) are used in the same ear, with the HI typically amplifying the residual low-frequency acoustic hearing. It has been shown that with EAS subjects the addition of low frequency acoustic stimulation often enhances speech understanding [6]. These findings, along with recently reported ways to encode low frequency information through electric stimulation [7, 8] motivated this work. We present an implementation and clinical validation of a sound coding strategy that transmits low frequency information for unilateral CIs.

One can convey low frequency information simply by extending the lowest cut-off frequency associated with the most apical electrode contact. However, the place-pitch percept for the most apical electrode contact will be higher than the frequency information mapped to it [7, 9]. For example Marel et. al [10] have shown that the HiFocus1J obtains a mean insertion depth of 480 degrees which corresponds approximately with a frequency of 480 Hz [11]. Adding additional low frequency information to the most apical electrode will result in further deviations from the subject’s spiral ganglion map. Some studies [12–14] suggest that implant users can adapt over time to spectrally shifted speech-frequency mappings. However, correcting the allocation of acoustic components to individual electrode contacts may be important for music and indeed for improved speech recognition [15–19].

**Fig 1:**
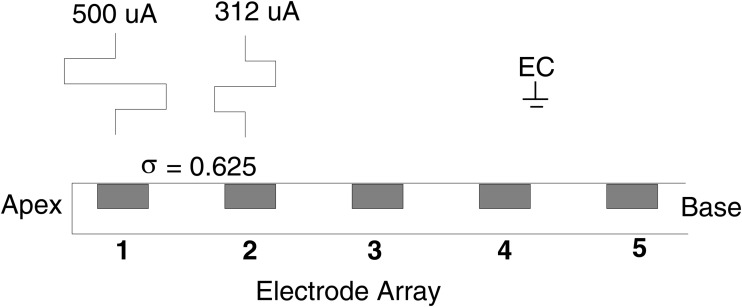
Schematic illustration of Phantom electrode configuration with primary electrode 1 and compensating contact 2 adapted from [7]. Here, the amount of current compensation is *σ* = 0.625.

**Fig 2:**
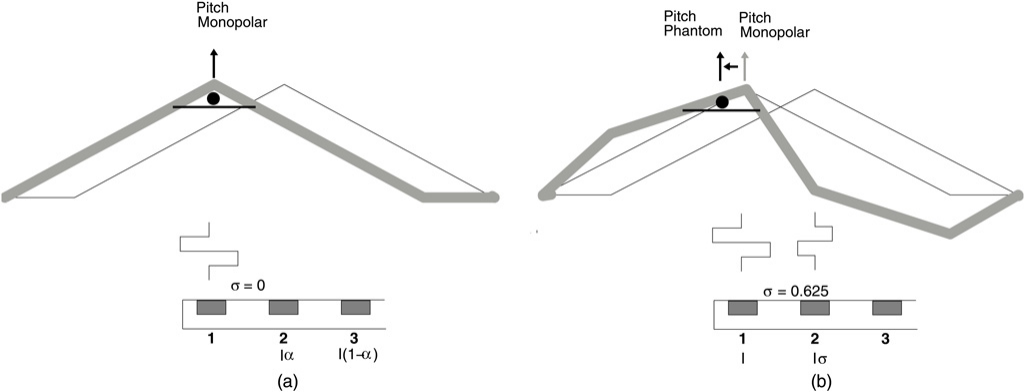
Schematic illustration of the Phantom effect for *σ* = 0 (a) and *σ* = 0.625 (b) on pitch perception. The electrical field is simulated using triangular functions and assuming linear superposition of the electrical field produced by each electrode. The center of masses of the electrical field is assumed to be related to the pitch elicited by the stimulation. Using Phantom stimulation it is possible to push the electrical field away from the most apical electrode.

Typically, monopolar electrode coupling is used to deliver electrical stimulation to the auditory neurons in today’s CI systems. Current flows between a primary intracochlear electrode and a remote extracochlear ground contact. For simultaneous current steering, stimulation is delivered to an adjacent pair of contacts using the same phase [20]. Shaping of the electrical field can be achieved by applying reverse phase compensating currents to the neighboring contacts. More recently, Saoji &; Litvak [7] presented a technique first reported by Wilson [21] based on biphasic pulses presented in partial bipolar mode. Saoji &; Litvak called this Phantom electrode stimulation. Phantom stimulation is illustrated in Fig. 1. Here a cathodic-anodic biphasic pulse was presented on the primary (apical) electrode contact 1, while a reverse phase pulse (anodic-cathodic) was presented on the compensating contact 2. The ratio of the current between the compensating and the primary electrode is termed *σ*. When *σ* = 0, all stimulation is on the primary electrode resulting in monopolar stimulation mode. When *σ* = 1, stimulation is equal between the primary and compensating electrode contact resulting in bipolar stimulation mode. When a partial return current (e.g., *σ* = 0.625) is presented on the neighboring basal contact, the spread of electrical excitation toward the high-frequency basal end of the cochlea is reduced [22], moving the electrical excitation more apically (Fig. 2). When Saoji &; Litvak [7] applied this technique to contacts in the middle of the electrode array, 10 Advanced Bionics cochlear implant users reported a lowering of pitch perception equivalent to 0.5 to 2 electrodes when compared to monopolar stimulation.

It has been suggested that accurate pitch perception may depend on a match between place and temporal cues, and that the mismatch between these two cues in CIs may limit discrimination performance in CI listeners [23–25]. The Advanced Bionics CI systems use the HiFocus1J electrode [26] which is typically inserted approximately 1.25 turns [10]. Using Phantom stimulation the insertion depth can virtually be increased by about 0.5 to 2 electrodes [7], which represents about 0.5 to 2 mm of additional insertion depth. In MED-EL CI systems the Standard and the FLEXSOFT electrode arrays are typically inserted approximately 1.75 to 2 full turns into the cochlea [27, 28]. Comparing the Standard and FLEXSOFT electrode arrays with the HiFocus1J, the most apical 1J electrode coincides approximately with the 3rd and 4th most apical electrode contacts of the MED-EL Standard electrode array. Using these long arrays Schatzer et al. [25] investigated pitch perception in a group of CI users with normal hearing in their non-implanted ear. They asked CI users to match rates of unmondulated pulse trains presented on one of their most apical six electrodes to pure tones at frequencies ranging from 100-450 Hz presented on their normal hearing ear. They found reliable electrical pitch percepts when rate and electrode place of stimulation were reasonably matched. Most subjects achieved pitch matches to pure tones up to 300 Hz only on electrodes at insertion angles larger than 360 degrees. Based on these findings they suggest that coding strategies that aim at representing low-frequency temporal fine structure via pulse rate modulations should map those fine-structure channels to electrodes placed in the second turn of the cochlea. However, it has also been shown [26, 29] that deeper insertion can lead to more insertion trauma increasing the possibility of some apical contacts translocating from scala media into scala vestibuli, with a negative impact on speech perception score. Phantom permits stimulation of the auditory nerve beyond the most apical electrode without using deeper electrode insertions, and therefore can be used to encode a lower frequency and to extend the range of stimulation sites and represented frequencies.

Using MED-EL Standard electrode arrays, Arnolder et al. [30] found that speech intelligibility can be improved increasing the electrode distance, and therefore decreasing channel interaction, in the apical part of the cochlea. It has been shown that Phantom stimulation produces a narrower electrical field than monopolar stimulation [22], and thus Phantom might be able to transmit electrical stimulation in the apical part of the cochlea more effectively.

Although some CI users can make use of temporal information at relatively high rate [31], the majority cannot perceive any difference for temporal modulations above 300 Hz [32, 33]. However, this rate limitation does not preclude coding of temporal envelope cues up to approximately 300 Hz, where rate or modulation rate of electrical pulse trains can be used to convey a percept of pitch [34]. Additionally some strategies like the FSP/FSP4 strategies used in MED-EL devices are intended for providing temporal fine structure (TFS) by using stimulations at the 1-4 most apical electrodes that are elicited at a variable rate that corresponds to the fine structure of the signal in the frequency range from 100 to 710Hz. It has been shown that transmitting fine structure in the low frequencies enhances speech perception in noise [35]. The Phantom channel is designed to convey temporal information on the most apical region of the cochlea. The idea is to transmit temporal fluctuations using high stimulation rates of around 1000 pulses per second to encode low frequency sounds from 65 Hz to 300 Hz.

Another pitch lowering technique has recently been proposed by Macherey et al [8]. Here pseudomonophasic pulses, consisting of short, high amplitude phase followed by a longer much lower amplitude opposite-polarity phase, are presented in bipolar mode. This work showed that rate pitch could be extended beyond the 300 Hz limit of monopolar stimulation when using pseudomonophasic pulses delivered to the most apical electrode contacts likely since neurons can phase lock to higher rates when neural information is originated from more apical sites of the cochlea. They also hypothesized that more focused stimulation in the cochlea, as provided by bipolar mode, is needed to convey better temporal information. Despite the above work, no sound coding strategy has yet been implemented with pseudomonophasic pulses in a commercial CI sound processor to allow chronic evaluation of speech intelligibility and music perception.

This study evaluates a new sound processing strategy that uses an additional Phantom channel to convey low frequency information in a slightly more apical region. The goals of this work were first, to investigate the applicability of such a strategy in a clinical sound processor; second, to investigate the fitting of the strategy; and third, to investigate whether the additional low frequency channel provided better speech intelligibility and sound quality than the clinical strategy.

### Materials and Methods

The Phantom strategy is based on the HiRes with Fidelity 120 (F120) strategy [36] from Advanced Bionics but adds an additional low frequency channel. To represent this new channel, a virtual electrode is created using partial bipolar stimulation (Phantom). Fig. 2 (left) shows the primary stimulating current being delivered from electrode contact 1. The electrical field produced by each electrode is modelled using a triangular function. In Fig. 2b an additional smaller compensating current with opposite phase is delivered from the adjacent contact 2. We assumed linear superposition of the individual electrical fields to simulate the overall electrical field created by Phantom stimulation. Using this simple model, it can be observed that the compensating current makes the working phase of the primary current less effective on the compensating (basal) side, resulting in an apical shift in field and hence neural recruitment. The Phantom channel thus provides a lower pitch sensation than that of the most apical electrode contact alone, making this channel suitable to convey low frequency information.

**Fig 3:**
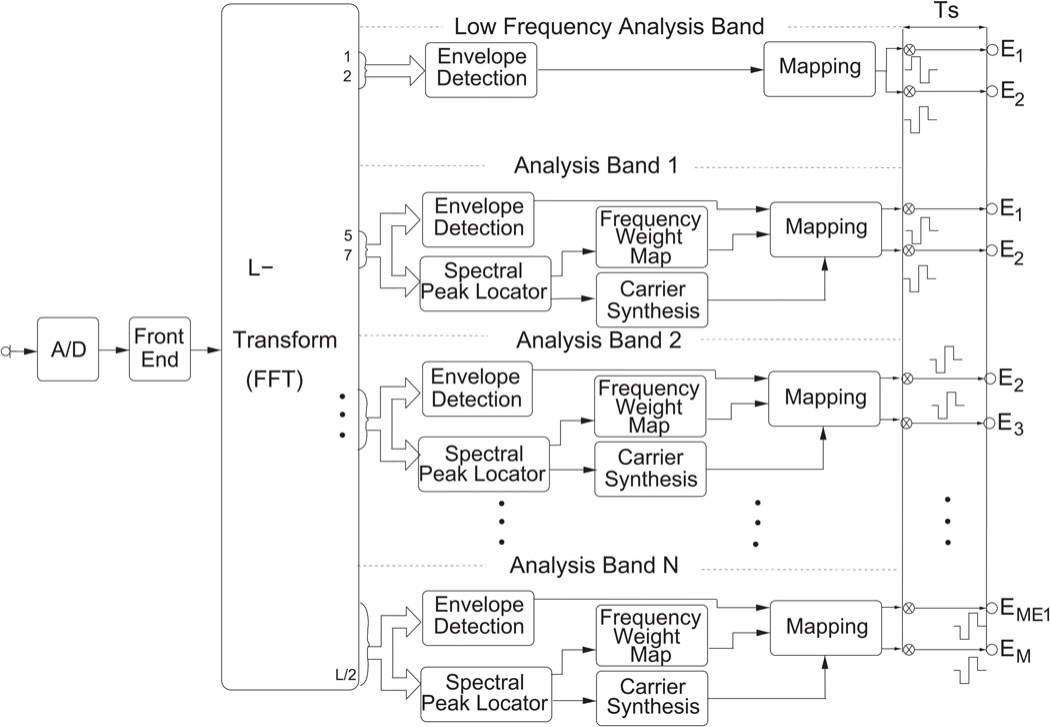
Block Diagram of the Phantom Strategy. The Phantom strategy incorporates a low frequency analysis band used to deliver information to a partial bipolar (Phantom) channel. The rest of the analysis bands are exactly the same as in the commercial F120 strategy.

Fig. 3 presents the basic block diagram of the Phantom strategy. The microphone signal is first digitized using a sampling frequency Fs of 17400 samples per second. Next the front-end implements a dual-action automatic-gain control (AGC) to remove the large variations in the acoustic environment [37, 38]. The resulting signal is sent through a filter bank based on a Fast Fourier Transform (FFT) of length L = 256 samples. The linearly spaced FFT bins then are grouped into 16 bands. Table 1 presents the number of FFT bins assigned to each analysis band and its associated center frequency. For each analysis band, the Hilbert envelope is computed from that channel’s FFT bins [36]. Non-linear amplification is used to compress the output of the envelope detector into the range between the threshold (T) level and most comfortable (M) level of a recipient’s electrical hearing range using a logarithmic compression function. For the lowest frequency analysis band, the envelope is used to amplitude modulate a pair of partial bipolar biphasic pulses like the one presented in the left side of Fig. 2. The partial bipolar channel is configured with a fixed value of *σ* that is set during the fitting session. The low frequency channel provides mostly temporal information because the spectral bandwidth associated with this channel is relatively large and no adaptive current steering is applied to this channel.

**Table 1:** Number of FFT bins related to each analysis band and its associated center frequencies in Hz. *z* is the band number, *N*_*z*_ is the number of bins in the *z* ^*th*^ band and *f*_*center*_ is the center frequency in Hertz.

**Band** *z*	**0**	**1**	**2**	**3**	**4**	**5**	**6**	**7**
**Bins** *N*_*z*_	4	2	2	1	2	2	2	3
*n*_*start*_*z*__	1	5	7	9	10	12	14	16
*f*_*center*_ **(Hz)**	170	408	544	646	748	884	1020	1190
**Band** *z*	**8**	**9**	**10**	**11**	**12**	**13**	**14**	**15**
**Bins** *N*_*z*_	4	4	5	6	7	8	10	55
*n*_*start*_*z*__	19	23	27	32	38	45	53	63
*f*_*center*_ **(Hz)**	1427	1700	2005	2379	2821	3330	3942	6491

**Fig 4:**
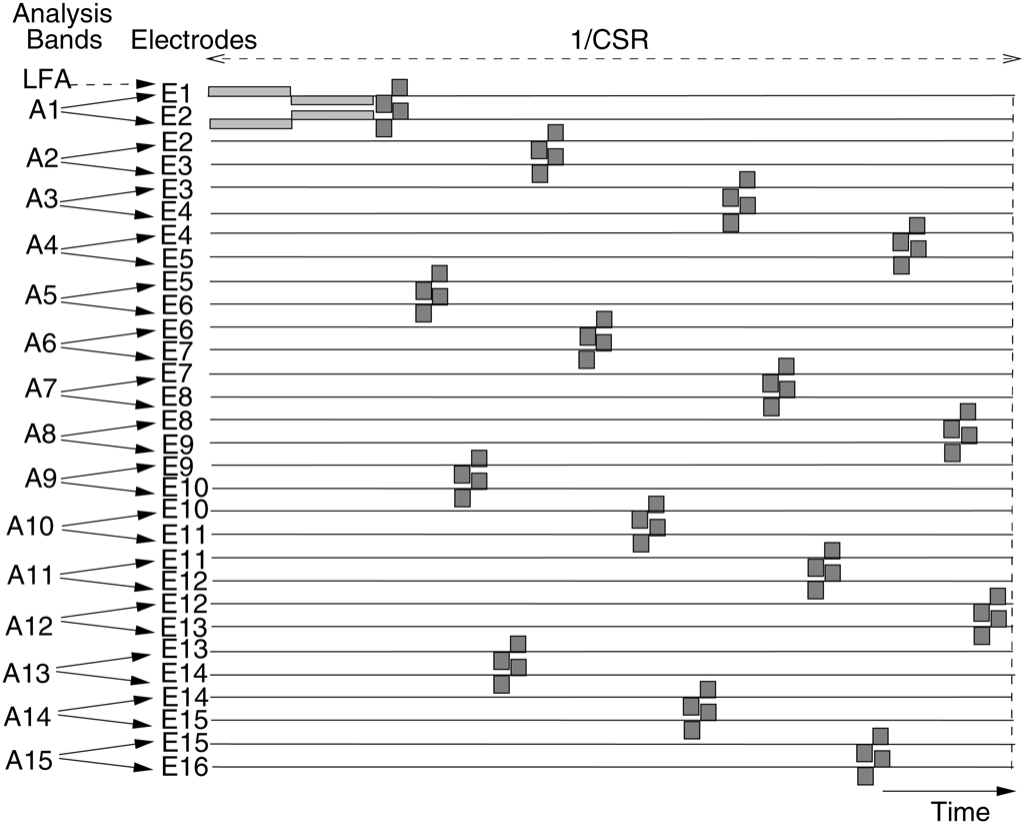
Stimulation pattern for one cycle of Phantom strategy stimulation. CSR is the Channel Stimulation Rate for one electrode. In the Phantom strategy, the electrode contacts 1 and 2 are stimulated simultaneously but out of phase prior to stimulating the rest of analysis bands using the standard in phase current steering technique.

For the remaining analysis bands, the standard F120 strategy processing is used [36]. For each analysis band the Hilbert Envelope is computed from that channel’s FFT bins (Table 1). Additionally, in order to improve the spectral resolution of the audio signal analysis, an interpolation is performed, based on a spectral peak locator within each analysis band. The spectral peak locator estimates the most important frequency in each analysis channel. A frequency weight map converts this frequency into a current weighting and carrier synthesis for the current steered electrode pair in each channel (see [36] for more details). For each stimulation cycle, the electrode pair associated with one analysis band is stimulated simultaneously. However, different channels are stimulated sequentially in order to reduce undesired channel interactions. Furthermore, the order of stimulation is selected to maximize the distance between stimulation pairs to further reduce channel interaction. Fig. 4 illustrates the stimulation pattern for a complete Phantom strategy stimulation cycle. Because partial bipolar stimulation requires a larger charge per phase to produce the equivalent loudness of monopolar stimulation, a longer phase duration is allocated to reduce the risk of stimulating at levels that are out of compliance. The Phantom phase duration was configured to be 6 times longer than that for the remaining electrode contacts, thus reducing the overall stimulation rate by 29% in comparison to a F120 strategy. No significant reduction in speech perception was expected from this rate reduction [39] given the relatively large stimulation rates used by the clinical strategies of the subjects (Table 2).

**Table 2:** Subject demographics.

**Patient id**	**Age**	**Duration of deafness in years**	**Cause of deafness**	**Implant experience in years**	**Electrode type**	**Stimulation Rate (pps)**
P1	69	0	genetic	6	HiRes90k	1736
P2	62	12.58	unknown	5.17	HiRes90k	1736
P3	34	0	otitis media	4.75	HiRes90k	1736
P4	60	3.25	sudden hearing loss	6	HiRes90k	1736
P5	76	0	suddden hearing loss	3.2	HiRes90k	1736
P6	49	3.75	sudden hearing loss	12.75	Clarion CII	1736
P7	32	0.75	unkown	13	Clarion CII	1201
P8	53	4	unknown	13.25	Clarion CII	1341
P9	61	5.75	unknown	6.17	HiRes90k	1736
P10	50	9.59	genetic	2.1	HiRes90k	1736
P11	50	30.58	sudden hearing loss	9.42	HiRes90k	1736
P12	56	0	unknown	11.67	Clarion CII	1024

The only difference between the Phantom and the subject’s clinical strategy (F120) is the addition of the low frequency channel (transmitted through Phantom stimulation) and the consequent reduction of stimulation rate. All other aspects of the strategy remain the same for both strategies.

### Subjects

The study protocol was reviewed and approved by a registered board (Freiburger Ethik-Komission International). Only adult CI users participated in the study. After explanation of the study protocol and the risks and benefits of participating, all subjects signed a consent form before participating. The consent form was approved by the ethics board.

12 postlingually deaf German speaking users of the Advanced Bionics CII or HiRes90k implants and the F120 strategy participated in the study. Only Advanced Bionics devices were used because of the need to simultaneously stimulate several electrodes in or out of phase, requiring multiple independent current sources. All subjects were postlingually deafened users and were able to score at ceiling on the HSM [40] sentence test delivered without background noise. All subjects had experience in previous clinical evaluations. Subject demographics, including age, duration of deafness and implant experience can be found in Table 2.

### Study Design

The Phantom and the commercial F120 strategy were evaluated in two sessions. In session one, impedances were measured for the 16 electrode contacts using a phase duration of 32.32 msec/phase. The amplitude of the test pulse was 16 *μ*Amperes. The impedance values were used to set the upper limit of the current (*μ*Amperes) that could be used to stimulate individual electrodes, assuming a maximum compliance voltage of 8 V for each current source. Also, a maximum charge density safety limit of 100 *μC*/*cm* ^2^ was used to ensure that the upper limit of the current (µAmperes) used to stimulate individual electrodes was within the safety limits applied for research studies by the United States Food and Drug Administration.

The Phantom strategy was fitted and stored on a Harmony research processor together with the F120 strategy. Next, speech tests based on the HSM sentence test were conducted to assess speech intelligibility with both strategies. The participants were instructed to use the Phantom strategy during the following 4 weeks.

### Fitting

The BEPSnet software (Advanced Bionics LLC) was used to fit both the F120 and Phantom strategies. The Phantom strategy was fitted based on the F120 clinical map, with only the fitting for the Phantom channel being different. Pulse trains at the same rate as used for the remaining channels, but with a phase duration 6 times longer, were delivered using partial bipolar stimulation to the two most apical electrode contacts. The Phantom channel fitting required two stages: 1) the most comfortable level for the Phantom channel (*M*_*ph*_) in *μ*Amperes was found and 2) the maximum value of *σ* that elicited a lower pitch sensation than electrode contact 1 in monopolar mode was found. Initially, Phantom was configured with a value of *σ* set to 0.625 because this value should be the one eliciting the lowest pitch perception while at the same time minimizing the risk of pitch reversal [7]. Given this value of *σ*, the initial M level on the Phantom channel (*M*_*ph*_) was set using the following empirical equation:

$$\begin{array}{c} M_{ph}=\frac{M_{EL1}}{(1-\sigma )N} \end{array}$$ (1),

where *M*_*EL*1_ denotes the M level on electrode 1 and N denotes the pulse width increase factor for the Phantom channel with respect to the remaining channels. Here N was always set to 6. Using *σ* = 0.625 means that 62.5% of the current flows from the primary electrode contact to the compensating contact and thus, does not contribute to loudness. Next, the level on the Phantom channel was modified until the subject perceived it to produce the same loudness sensation as electrode contact 1 stimulated at *M*_*EL*1_ in monopolar mode.

Later the subject was asked whether Phantom sounded lower in pitch than electrode contact 1 (using monopolar stimulation). The BEPSnet software allowed us to change contact 1 back and forth between Phantom and monopolar mode. The subject was asked to identify the lowest pitch sensation from the two stimuli (electrode 1: monopolar or Phantom). This task was repeated four times in a randomized order with the subject blind to the stimulation mode. If a subject selected Phantom to sound lower in pitch 100% of the time, that value of *σ* was allocated. Otherwise the value of *σ* was reduced to 0.5, the *M*_*ph*_ was reestimated and the experiment repeated until Phantom was perceived to sound lower in pitch than electrode 1 in 100% of the trials. Finally, the strategy was switched on and the subject was asked about the sound balance produced by Phantom. If a sound quality was too much dominated by low frequency, the *M*_*ph*_ was slightly reduced.

### Speech Tests

Each condition was evaluated using two lists of the HSM-Sentence test [40]. Sentences were mixed with speech shaped CCITT noise (according to ITU-T Rec. G. 227 11/88 Conventional telephone signal) [41]. The Signal-to-Noise-Ratio was selected individually depending upon the performance of each individual subject. Testing was conducted in a sound treated room. The HSM sentences were played through a loudspeaker placed at 0 degrees azimuth using a presentation level of 65 dB(A). The distance between the participant and the loudspeaker was 1 meter. The HSM sentence test uses balanced lists of 20 sentences with a fixed SNR. The performance is measured in% of correct words correctly repeated. After completion of the chronic phase (4 weeks), the subject returned for a second session, at which time two HSM lists were presented with both programs (Phantom and F120).

### Music Perception

At the end of the first session, the CI users listened to different musical pieces and were asked about their impression of music while using Phantom and F120. The first music piece was composed of 3 sentences of the Orchestersuite Nr. 2 H-Moll (BWV 1067) of Johann Sebastian Bach, where the section without Basso Continuo was removed (it was assumed that this passage should sound very similar using both strategies). The second music piece was Serendepity by the Jazz/Fussion-Bass player Tal Wilkenfeld, which contains a strong rhythm and a lot of low frequency content. The music pieces were presented through a standard loudspeaker and a subwoofer at 0 degrees azymuth to ensure that low frequency components were properly transmitted. The subwoofer was callibrated to present the music pieces at the same level of 60 dB SPL as the other loudspeaker at a 1 meter distance. For the second session, the selection of the music pieces was added to with the music piece “We only get what we give” from the “New Radicals”. This was a typical song from the Rock/Pop genre and the music piece contains vocals. Using these music pieces we developed a music questionnaire, based on the questionnaire from [42], that allowed for a direct comparison of the two programs. The questionnaire was filled only at session two. The following questions were asked, while the music piece between brackets was played:

- How easy is to follow music? (Music piece: New Radicals)
- How natural does the music sound? (Music piece: Bach)
- How good/natural is the tonal balance of the music? (Music piece: Tal Wilkenfeld)
- What is your overall impression of music? (All music pieces)
- With which program do you prefer to listen to music?

The first 4 questions were presented using a 5 step scale, each step contained 3 sub-steps, thus creating a 15-step Likert scale [43]. For example, the possible responses to question 2 were: very natural, natural, neutral, unnatural, very unnatural. Additionally, each step was accompanied with pictures to help convey their meaning. The subjects heard around 20-30 seconds of each music piece with each program. The program was changed without the subject knowing which strategy corresponded to which program. The goal of question 1 was to probe the intelligibility of sung music, as well as the ability to hear to each instrument group. Question 2 was asked to assess the quality and natural presentation of each instrument. Question 3 asked about the sound balance perceived with each strategy (from very low or “bass” like to very high or “treble” like). To answer question 4 all the music pieces were played again with each program. The final question was a preference between F120 or Phantom for listening to music, taking into consideration only the pieces presented during the questionnaire.

## Results

### Fitting of Phantom Channel

**Fig 5:**
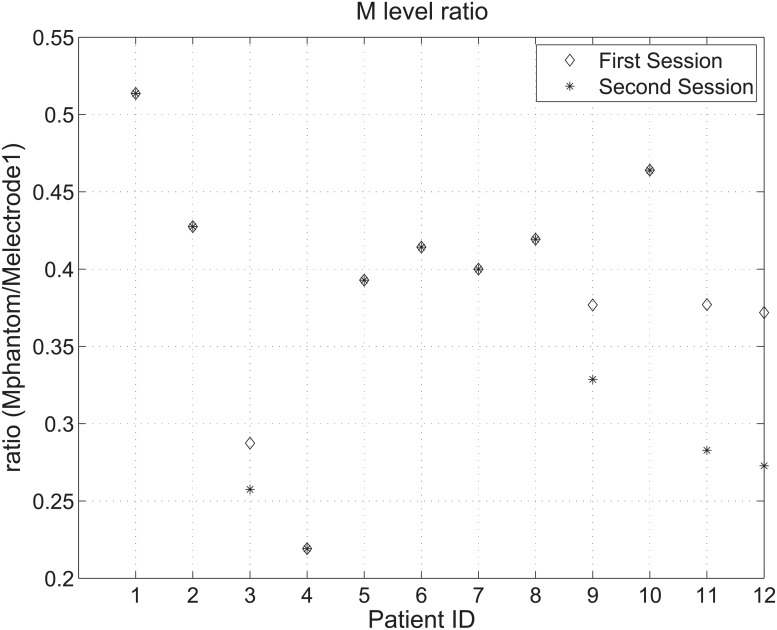
Ratio between M level on Phantom (*M*_*ph*_) and M level on electrode 1 (*M*_*EL*1_) for the first (diamonds) and second (stars) sessions. Study participants 3, 9, 11 and 12 were refitted during the second session reducing the *M*_*ph*_ level.

The fitting of the Phantom strategy was successful for all participants, meaning that all subjects were able to use Phantom in their daily life during a 4 week period. With the initial setting of *σ* = 0.625, 9 of 12 participants found that the Phantom channel sounded lower in pitch than electrode 1 in monopolar mode. The value of *σ* and the corresponding M level for each participant are presented in Table 3. For two subjects, we hypothesized that the Phantom channel caused a pitch reversal and for this reason, the value of *σ* was reduced. Subject ID 4 could not perceive a lower pitch sensation when using Phantom with any value of *σ* and therefore was eliminated from the study. This case shows that there may be a small proportion of patients for which Phantom is inappropriate as a strategy. The ratio between the M level on Phantom *M*_*ph*_ and the M level on electrode 1 for each participant is presented in Fig. 5. The description of the sound produced by Phantom was very different among participants. One subject could not perceive any difference between the two programs, while the remainder were surprised by the dominant low frequency sound. For example, participant ID 1, who was fitted with a very high M level ratio between electrode 1 and Phantom, described the sound to be massively dominated by the low frequencies. For the remaining participants, the M level on the Phantom channel was slightly reduced after switching on the strategy, in order to get a better balance between high and low frequencies. From the informal music test at the end of the first test session, most of the subjects reported that they liked the bassy sound produced by the Phantom strategy to listen to music.

**Table 3:** Value of *σ* and corresponding M level on the Phantom channel for session 1.

**ID**	**1**	**2**	**3**	**4**	**5**	**6**	**7**	**8**	**9**	**10**	**11**	**12**
***σ***	0.625	0.625	0.625	0	0.625	0.625	0.625	0.5	0.5	0.625	0.625	0.625
**M level**	208	336	192	174	374	280	264	608	188	264	440	180

At the beginning of the second session, the participants were again asked about their impression when listening to both programs during the 4 week take home trial. Here again there was a divided opinion. Around half of the participants responded positively about experience with the Phantom program for speech and music. In general, they were satisfied with the improved sound quality and the more natural sound perception. 6 participants showed a strong preference for Phantom, and they reported that they would like to use this strategy as a main program. The other 6 participants showed no preference or were even dissatisfied with Phantom, mostly because the Phantom channel sounded too loud. The sound was described as unclear and with echo on their own voice. However, these subjects did not report any negative effect from Phantom during the fitting phase, probably because they were fitted in a studio room with low noise. For these subjects, the *M*_*ph*_ levels were reduced for the Phantom electrode. After this refit (Tables 3 and 4), all these subjects obtained an improved sound quality.

**Table 4:** Value of *σ* and corresponding M level on the Phantom channel for session 2.

**ID**	**1**	**2**	**3**	**4**	**5**	**6**	**7**	**8**	**9**	**10**	**11**	**12**
***σ***	0.625	0.625	0.5	0	0.625	0.625	0.625	0.5	0.5	0.625	0.625	0.625
**M level**	208	336	172	174	374	280	264	608	164	264	330	132

### Speech Understanding

**Fig 6:**
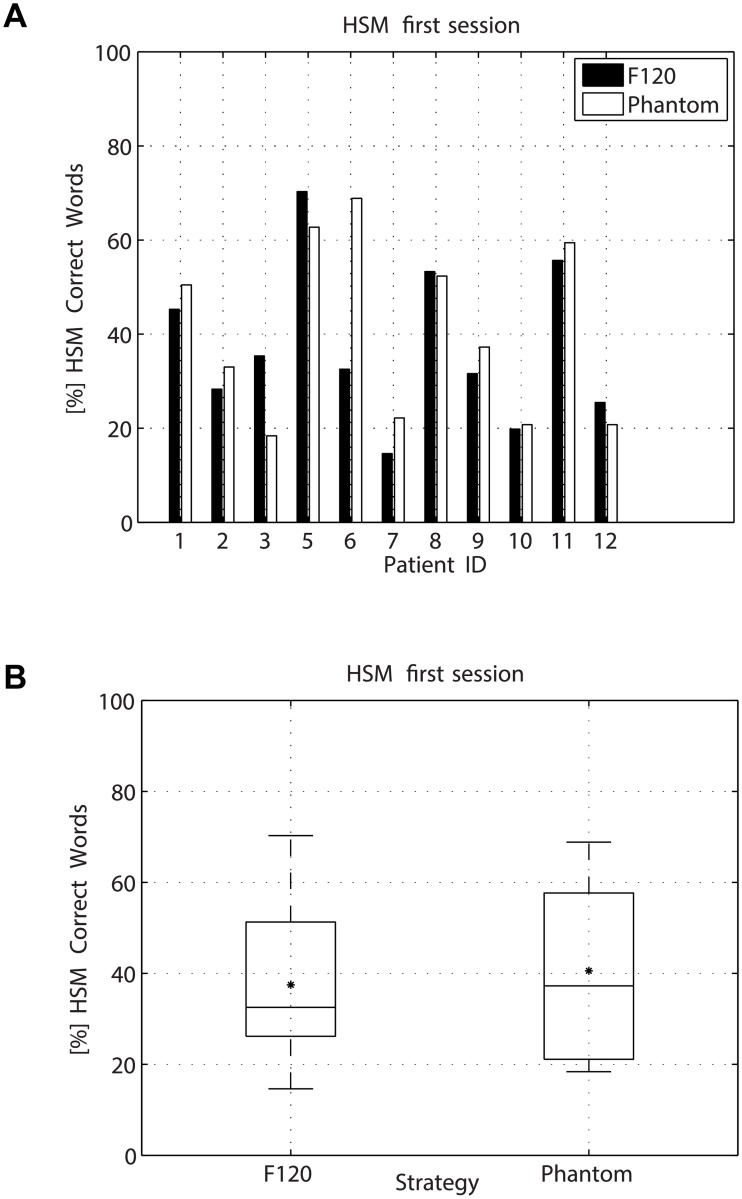
Results for the HSM sentence test during the first session. No significant difference was observed (paired t-test p = 0.455). The horizontal line in the box indicates the median value and the asterisk indicates the mean value.

**Fig 7:**
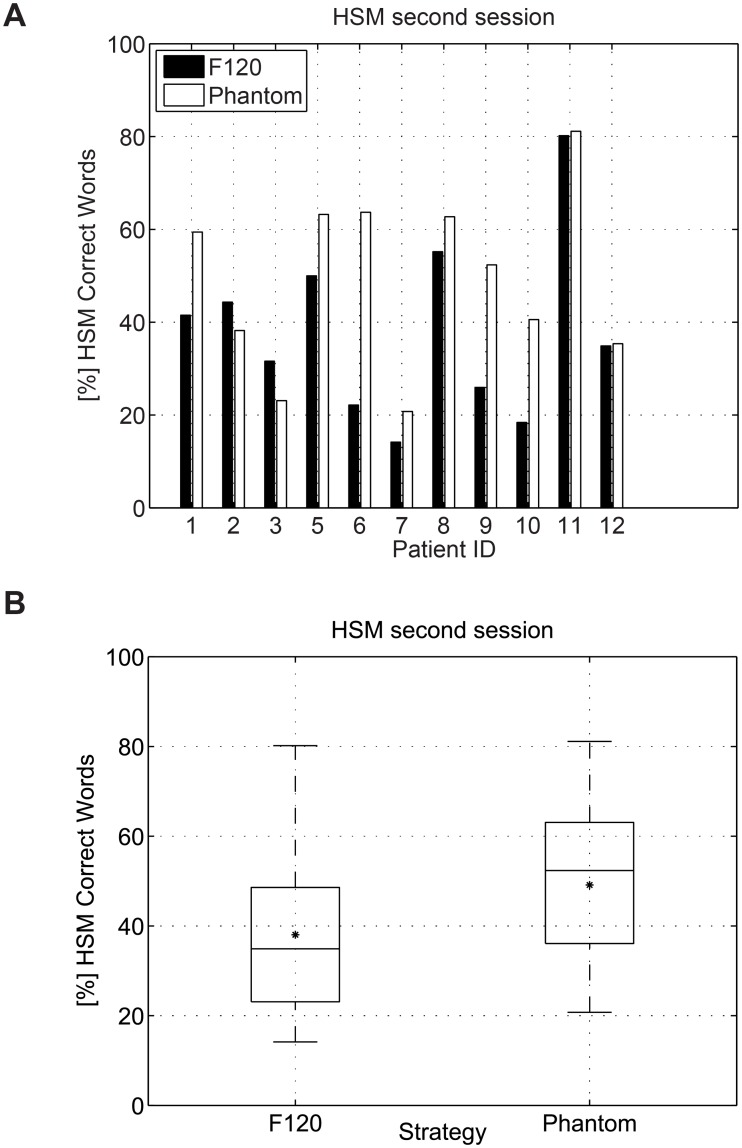
Results for the HSM sentence test during the second session. No significant difference was observed after Bonferroni correction (paired t-test p = 0.034).

**Fig 8:**
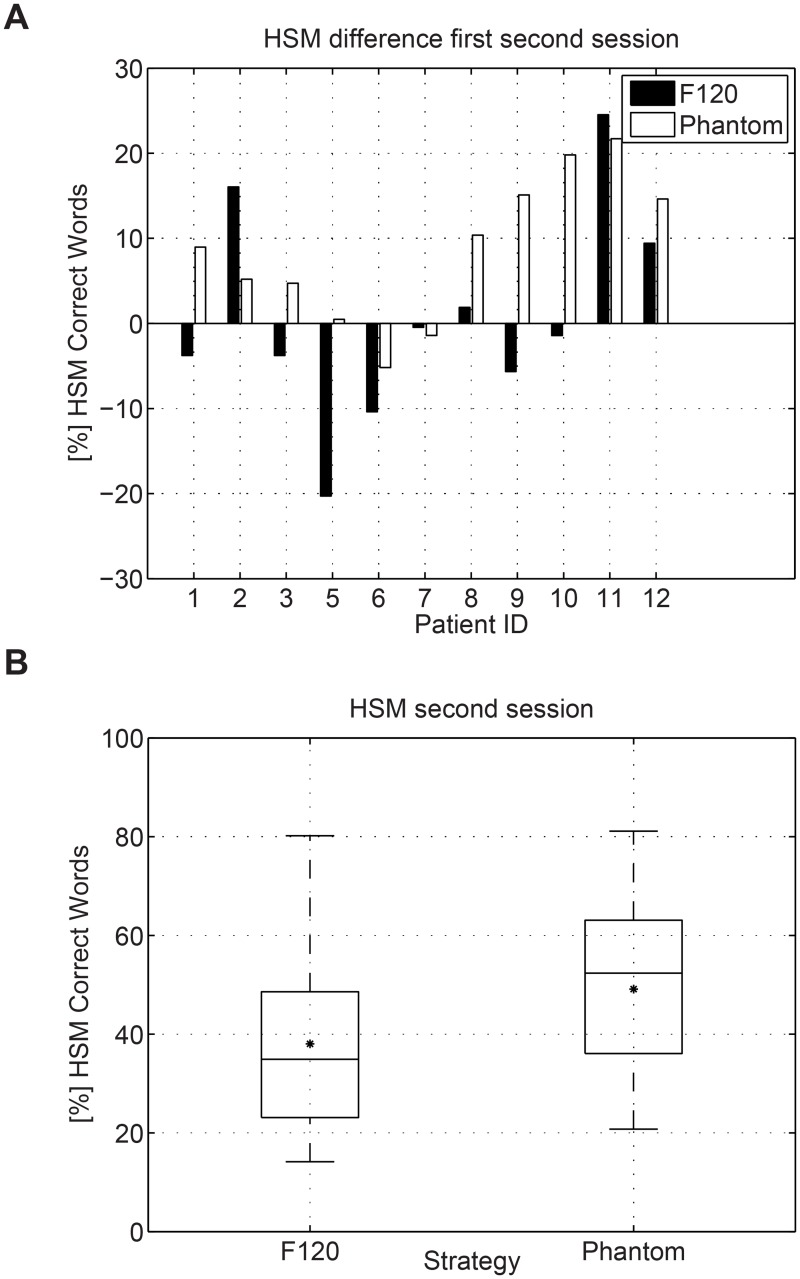
Difference in HSM performance between the first and the second session. A repeated measures of ANOVA within factors strategy (F120, Phantom) and interaction time (Session1, Session2) revealed a significant effect for the interaction time and strategy [F(1.00) = 6.476; p = 0.029].

Speech perception was evaluated using the HSM sentence tests in noise using both, the F120 and the Phantom strategy. The sentence test was performed at a SNR of 5, 10 or 15 dB depending on the performance of each participant. The amount of noise was selected such that the performance in% of correct words remained between 25% and 75%. Table 5 shows the SNR level at which the HSM sentence test was performed. The results for session 1 and 2 are presented in Figs. 6 and 7 respectively. Fig. 8 presents the difference in HSM speech performance between session 1 and 2 for each strategy. In the Figures, the median value is indicated by a horizontal line and the mean value is indicated by an asterisk. A repeated measures of analysis of variance (ANOVA) within factors strategy (F120, Phantom) and interaction time (Session1, Session2) revealed a significant effect for the interaction time and strategy [F(1.00) = 6.476; p = 0.029]. Post-hoc paired t-tests were type I error corrected using Bonferroni correction which requires p < 0.0125. For the first session no significant differences between F120 and Phantom were observed, the mean value for F120 and Phantom was 37.47% and 40.56% respectively (paired t-test t(10) = 0.777, p = 0.455). For the second session, no significant difference between Phantom and F120 was observed after Bonferroni correction, although it seems that there is a trend towards better performance for Phantom (48.07%) with respect to F120 (36.96%) (paired t-test t(10) = 2.449, p = 0.034) after 1 month of use. Phantom obtained a significant improvement in speech intelligibility from the first session to the second session (40.56% vs 48.07%, paired t-test t(10) = 3.270, p = 0.008). No significant differences were observed between session 1 and 2 for F120 (37.47% vs 36.96%, paired t-test t(10) = 0.163, p = 0.874).

**Table 5:** SNR used to evaluate the HSM sentence test.

**ID**	**1**	**2**	**3**	**5**	**6**	**7**	**8**	**9**	**10**	**11**	**12**
**SNR (dB)**	10	5	15	10	5	10	10	10	10	5	10

Participant ID 6 obtained a remarkable improvement when using the Phantom strategy. This participant reported that using Phantom, he could perceive much better the intonation of the voices and this was giving him the possibility to understand speech better.

### Music Perception

**Fig 9:**
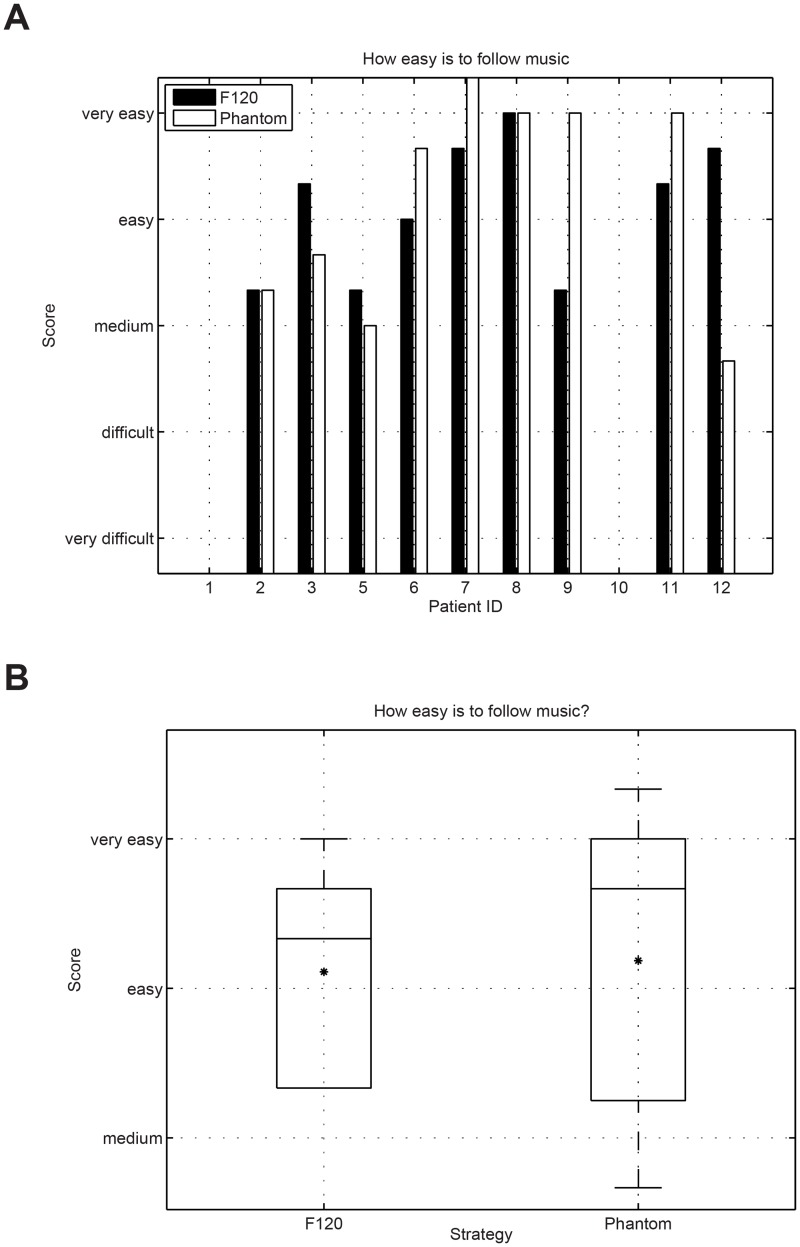
Results for the question “how easy is to follow the music”. No significant difference was observed between F120 and Phantom (wilcoxon signed tank test = 0.886).

**Fig 10:**
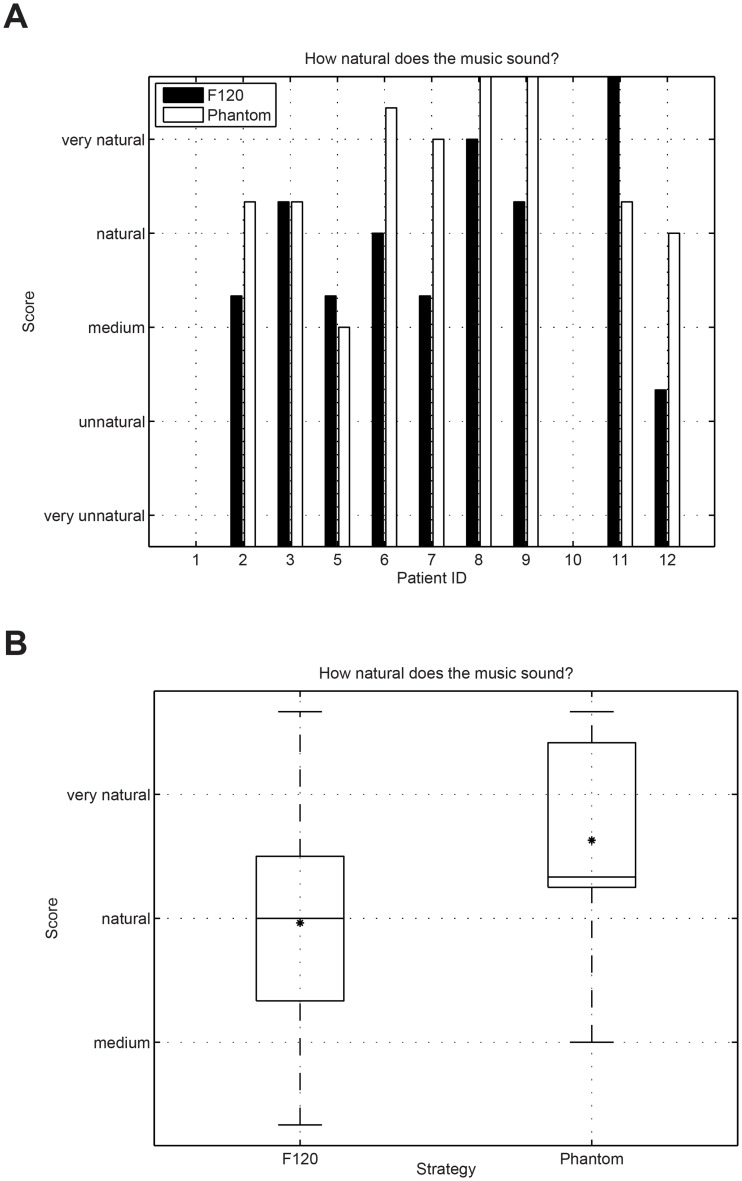
Results for the question “how natural does music sound”. No significant difference could be observed between both strategies (wilcoxon signed rank test p = 0.091).

**Fig 11:**
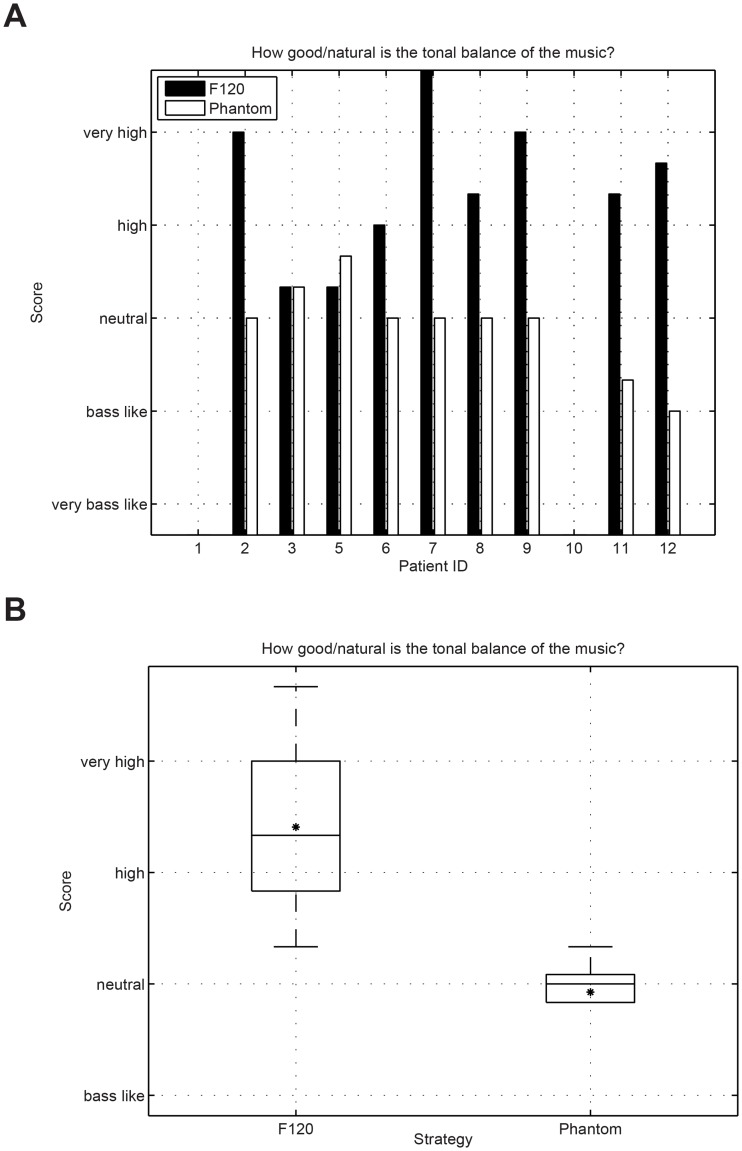
Results for the question about the “sound balance”. Phantom produces a significantly (wilcoxon signed rank test p = 0.01) more ballanced sound between the low and high frequencies than F120.

**Fig 12:**
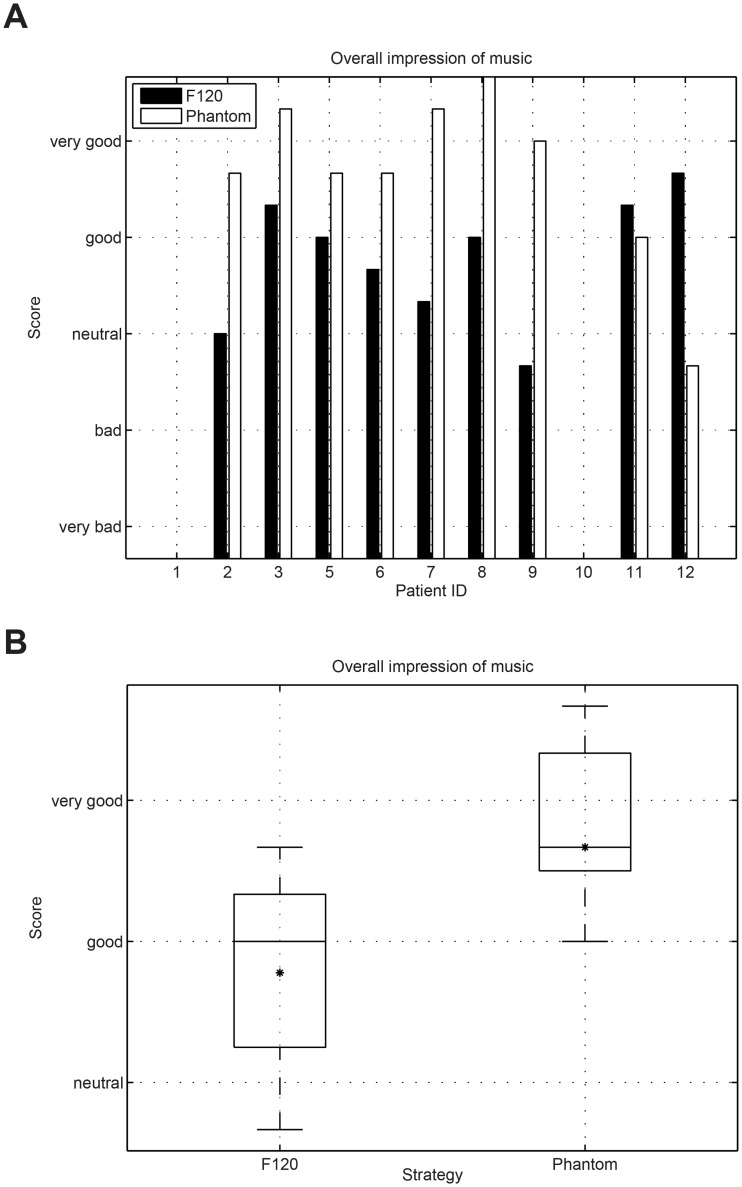
Results for the question about the “overall impression” of music. The Phantom strategy was rated to sound significantly better than F120 (wilcoxon signed rank test p = 0.037).

Music was assessed in a controlled comparison condition via our own questionnaire. Study participants ID1 and ID10 were not selected to conduct the music questionnaire because they were not able to perform reliable music assessments. The responses were analyzed using a wilcoxon signed-rank test to assess their significance. Fig. 9 presents the results for the question “how easy is to follow the music”. No significant difference between F120 and Phantom could be observed for the question “easy to follow” (wilcoxon signed rank test p = 0.886). Some participants reported that because they were used to the sound produced by F120, it was easier for them to follow the sound using the F120 strategy (only 4 weeks of accommodation time using Phantom). Fig. 10 presents the results for the question “how natural music sounds”. No significant difference could be observed between both strategies (wilcoxon signed rank test p = 0.091). Fig. 11 shows the results for the question about the perceived sound balance. The results for F120 are situated above the natural region, whereas Phantom was rated much lower in balance than F120. This question revealed that Phantom sounds significantly more balanced than F120 (wilcoxon signed rank test p = 0.001). Fig. 12 shows the results for the question about the overall impression of music. The overall impression of music with Phantom was rated higher than with F120. This difference was significant (wilcoxon signed rank test p = 0.037) and shows the overall preference of the CI users to listen to music using the Phantom strategy as presented in Table 6.

**Table 6:** Results of the preference question to listen to music.

**Preference**	**F120**	**Phantom**	**Sum**
**Number of listeners**	1	10	11
**Percentage**	9.09%	90.90%	100%

The correlation between the differences (F120-Phantom) in speech intelligibility and the responses of the music questionnaire were evaluated for statistical significance by means of Spearman’s rank order correlation coefficients. No significant relationships were found.

## Discussion

This study has presented a comparison between the clinical F120 strategy and a new strategy termed Phantom that adds an additional low frequency channel. The low frequency channel transmits frequencies below 300 Hz and presents them to the auditory nerve by stimulating the two most apical electrodes using a partial bipolar configuration. The low frequency channel aims to convey mostly temporal information to a region of the cochlea that is more apical than the most apical physical electrode contact. Research in the field of combined electric and acoustic hearing (EAS) has shown that low frequency information (below 300 Hz) perceived through the residual hearing can improve speech intelligibility and sound quality in general [6]. Based on this finding we investigated whether the coding of low frequencies can be improved through electrical stimulation only. The Phantom strategy was implemented in a commercial Harmony processor which allowed us to investigate the fitting procedure, as well as the perception of speech and music in a 1 month take home trial. The participants were selected from the database of the Medical University Hannover. These users were selected because of their good performance with their cochlear implant (near 100% speech intelligibility in the HSM sentence test without background noise), allowing a meaningful subjective feedback when comparing the sound sensations with both strategies.

### Fitting

The subject’s impression of Phantom while listening in quiet was primarily used to create a fitting. The fitting of the Phantom channel is challenging because small changes in the M level produce large effects on the overall sound perception. One reason for this is that the new channel is used to transmit around two additional octaves on the low frequency region which the subjects have not been able to perceive since their implantation. In this study, a fitting procedure has been proposed. First for each subject the optimal value of *σ* has to be determined; where optimal *σ* is defined as the *σ* that produces a maximum pitch shift. For that value of *σ* the corresponding M level has to be fitted. Second, the M level on Phantom is fitted such that it elicits the same loudness perception as the loudness perceived when electrode 1 is stimulated in monopolar mode at its M level. An empirical equation to match the amplitude of the Phantom channel as a function of *σ* has been used to set the initial value for the fitting. However, many participants reported that using this value, the Phantom strategy sounded too loud and was dominated by low frequencies when all the channels were activated. For this reason, the M level on the Phantom channel was slightly reduced to optimize the sound balance between the low and high frequencies.

#### Effects of Rate

The new partial bipolar channel was configured to use a longer pulse width (6 times longer) than the rest of channels. This caused the Phantom strategy to produce a 29% slower stimulation rate in comparison to F120 (Table 2), which in theory, can affect negatively the temporal information on each electrode. This aspect needs to be further investigated in a follow-up study. This configuration was chosen in order to reduce the amount of current needed to produce a comfortable loudness sensation on the Phantom channel at the expense of reducing the overall rate of the strategy. A benefit of the longer pulse width is that the Phantom strategy did not cause any additional power consumption on the device.

#### Effects of increasing bandwidth

Phantom can be considered as a 16 electrode strategy with an additional apical electrode. We observed clear differences in speech and music perception. With a correct fitting, as those used during the second session, most of the subjects perceived a fuller and more natural sound. Using Phantom, music perception was rated to sound significantly better than using the clinical strategy, most probably because the overall sound balance was rated to sound significantly more neutral when using the Phantom strategy. We think that the optimization of the fitting procedure can have a positive impact on speech intelligibility in noise as observed with participant ID 6.

One could argue that the improvements observed in Phantom are produced by the increased low frequency bandwidth transmitted by this strategy. Vermeire et al. 2010 [35] reported that an extended low frequency bandwidth does not provide with a significant improvement in speech intelligibility in noise. However, they could show a significant improvement in speech intelligibility when adding temporal fine structure in the extended low frequency spectrum as provided by the FSP strategy through very apical stimulated electrodes. Unlike the study of Vermeire et al. 2010, the Phantom strategy does not transmit temporal fine structure explicitly and all subjects participating in the Phantom study received a functionally deeper insertion than they were used to. We think that the small shift in apical stimulation provided by an additional Phantom channel might be the reason why CI users seem to obtain benefits in speech intelligibility and music perception.

Using Phantom we could not observe a significant difference in speech intelligibility with respect to F120, although the data seems to show a trend towards better performance with Phantom and some CI users obtained clear benefits from the strategy. It is possible that differences in performance are just caused by the addition of frequencies below 300 Hz. However, in a study of Vermeire et al. [35] it was reported that low frequency bandwidth does not provide with a significant improvement in speech intelligibility in noise. Actually, they could show a significant improvement in speech intelligibility when adding temporal fine structure in the low frequencies through deeply inserted electrodes. Therefore, it seems that the small shift in apical stimulation produced by Phantom, or the fact that the stimulation is provided with an extra channel might be the cause to explain the improvements observed in individual CI users.

### Speech Tests

Overall the speech intelligibility performance of the participants was very good at SNRs of 5 and 10 dB. In this study, it was not possible to show a significant improvement in speech intelligibility for Phantom with respect to the commercial strategy. The HSM sentence test was presented in noise at a fixed SNR that was adapted to the performance of each participant. For each condition, 2 HSM lists were presented. For the first session, the mean scores for F120 and Phantom were 37.47% and 40.56% respectively (paired t-test t(10) = 0.777, p = 0.455). For the second session Phantom showed a trend towards an improvement in speech intelligibility with respect to the F120 strategy, however results were not statistically significant after Bonferroni correction (36.96% F120 and 48.07% Phantom t(10) = 2.449, p = 0.034). The performance with Phantom increased significantly by almost 8% after one month of use (paired t-test t(10) = 3.270, p = 0.008). We think that speech intelligibility with Phantom could be further improved by increasing the take home period because other studies have shown increasing performance in speech intelligibility even three months after using a low frequency strategy for the first time [35, 44].

### Music Questionnaire

Music perception obtained by CI users is limited by the poor pitch perception obtained with these devices which causes difficulties in instrument identification, melody recognition and harmonicity [45–47]. However, results from recent studies have shown that CI users perceive music well enough for making reliable subjective comparisons [44, 48]. In this study, a novel questionnaire has been presented that allows a direct comparison between two programs. CI subjects reported enjoyment during the execution of the questionnaire. It seems that CI technology allows for music enjoyment and for this reason, we think that more effort has to be made to create new strategies specially designed to improve music perception.

Additionally, the music questionnaire has shown that the sound balance with Phantom was significantly more neutral than with the clinical F120 strategy. It appears that in general music with CIs sounds too high pitched. This result is supported by [49] who suggest to enhance M levels on the low frequencies to improve music perception in CIs. In our study, the sound balance was compensated by introducing the Phantom channel and this might explain, at least partially, the significant preference of the participants for listening to music with Phantom. However, it remains unclear whether music perception is preferred with Phantom because this strategy makes better use of the temporal pitch mechanism, which is available until least 300 Hz. Further research is needed to understand better the mechanism that provides improved music perception with Phantom.

The music questionnaire has been shown to be a successful method to assess the sound quality differences between Phantom and F120. In two categories of the music questionnaire it was possible to show a significant result. Furthermore, the execution of the music questionnaire was fluent and pleasant for the participants. We think that for future studies the use of subjective questionnaires with founded questions can be very useful for the evaluation and development of new strategies.

### Outlook

The Phantom strategy introduces a new low frequency channel and extends the low frequency bandwidth transmitted. A side effect of the addition of the low frequency channel is an overall reduction of stimulation rate. According to previous studies, no significant effect on performance is expected by this moderate reduction in stimulation rate. A follow-up study should investigate which of these factors has more impact on the promising trends in performance provided by Phantom. Psychophysical experiments should give more insight about the functioning of the Phantom channel. For example, the possible benefits for pitch perception using more apical stimulation and its correlation to music and speech perception should be more deeply investigated.

The design of the Phantom strategy has to be further optimized. So far, the Phantom channel has been used to transmit low-pass filtered sound signals below 300 Hz. The cut-off frequency that gives best performance should be investigated. Another topic of research is the type of information that has to be transmitted through this channel. If the fundamental frequency would be the cue that is causing the potential improvements when using Phantom, possibly only the fundamental frequency should be transmitted through the low frequency channel. However, there might be other cues, such as amplitude modulations of the fundamental frequency, or onsets and offset of sounds that might also help perceive sound. Additionally, the Phantom channel is currently used to transmit a relatively wide bandwidth of 2 octaves. The bandwidth allocated to this channel is much larger than that allocated to the remaining channels. We hypothesize that a possible improvement for Phantom could be the creation of additional Phantom channels using different values of *σ*. Each Phantom channel could be used to transmit different low frequency bands to not only make use of the temporal information, but also make use of place information in this low frequency region. However, additional Phantom channels would interact with each other because of the current spread in the cochlea and this fact could smear the temporal information delivered by a unique Phantom channel. To solve this issue one could use channel compensation techniques to reduce the negative effects of channel interaction [50].

## Conclusion

The clinical F120 strategy and a new strategy termed Phantom which is identical to F120 except that it includes an additional channel to transmit low frequencies were evaluated in 12 CI users in a 4 week take home trial. The fitting of the Phantom channel was crucial to obtain good sound quality. 11 out of 12 CI patients obtained a pitch perception with the partial bipolar (Phantom) channel lower than the pitch perceived when stimulating electrode 1 in monopolar mode. Speech performance with F120 and Phantom were evaluated immediately after fitting Phantom for the first time and after one month of take-home experience. No significant differences could be observed between the group mean performances with Phantom and F120. Moreover, no significant difference could be observed between both strategies for the questions “how easy is to follow music” and “how natural does the music sound”. However, the sound produced by Phantom was reported to be significantly more neutral than with F120. This result probably explains why the overall impression of music was rated higher when listening with Phantom and the significant preference for Phantom to listen to music.
